# Predicting 6-month mortality of patients with traumatic brain injury: usefulness of common severity scores

**DOI:** 10.1186/cc13669

**Published:** 2014-03-17

**Authors:** MB Skrifvars, R Raj, S Bendel, T Selander, R Kivisaari, J Siironen, M Reinikainen

**Affiliations:** 1 Helsinki University Central Hospital, Helsinki, Finland; 2 Kuopio University Hospital, Kuopio, Finland; 3 North Karelia Central Hospital, Joensuu, Finland

## Introduction

Severity of illness scoring systems is paramount for the evaluation of quality of care of critically ill and trauma patients [1-3]. The purpose of the present study was to evaluate the usefulness of the Acute Physiology and Chronic Health Evaluation II (APACHE II), Simplified Acute Physiology Score II (SAPS II) and Sequential Organ Failure Assessment (SOFA) scores in predicting long-term outcome of patients with moderate-to-severe traumatic brain injury (TBI).

## Methods

A Finnish multicenter ICU database was screened for TBI patients admitted in 2003 to 2012. Logistic regression was used for customization of the APACHE II, SAPS II and SOFA for 6-month mortality prediction. An adjusted SOFA model, including age, and a reference model, including only age and Glasgow Coma Scale, were built for comparison. A randomized split-sample technique was used for internal validation and prognostic performance was determined by assessing discrimination, calibration and precision.

## Results

**Figure 1:**
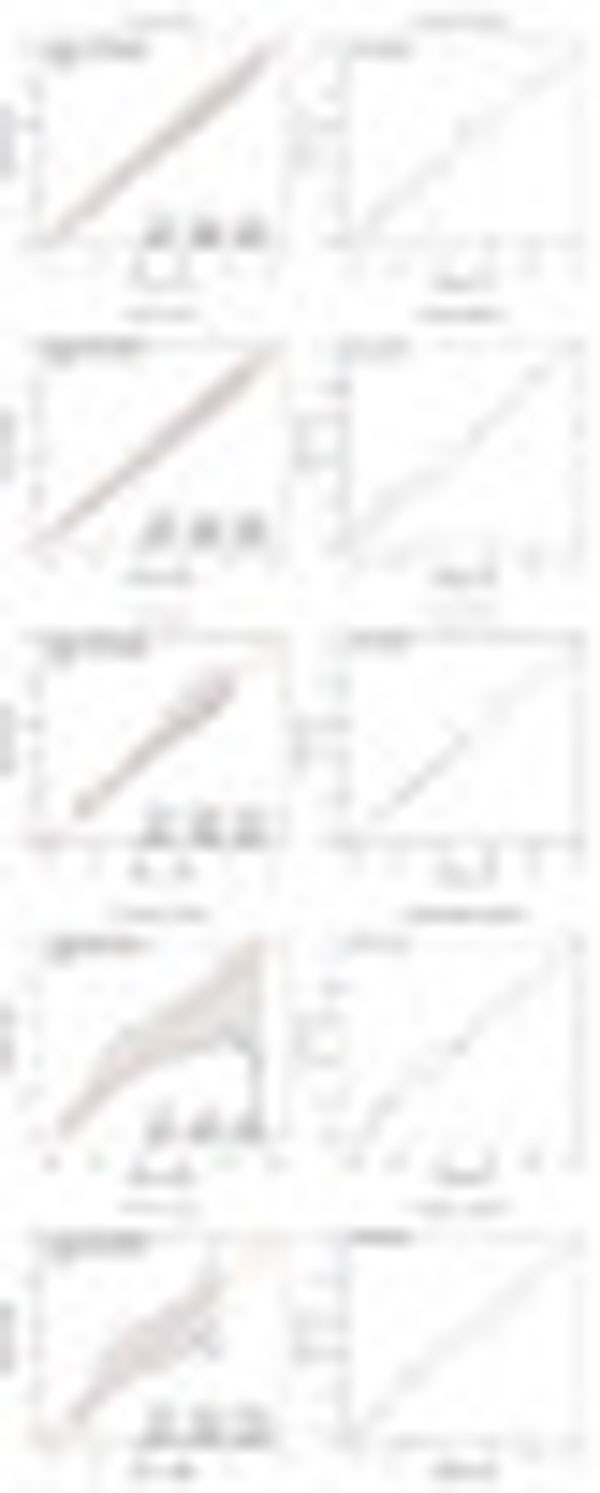
Calibration.

**Figure 2:**
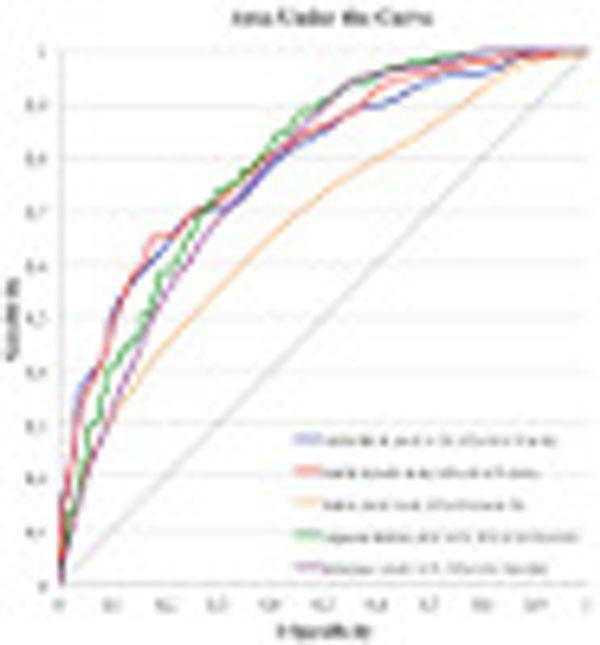
Discrimination.

A total of 1,625 patients were included. Overall 6-month mortality was 33%. The APACHE II and SAPS II-based models showed good discrimination (area under the curve (AUC) 0.79, 95% confidence interval (CI) = 0.75 to 0.82; and 0.80, 95% CI = 0.77 to 0.83), calibration (P >0.05) and precision. The SOFA-based model showed poor discrimination (AUC 0.68, 95% CI = 0.64 to 0.72) and precision but good calibration (P >0.05). The adjusted SOFA model displayed better discrimination (AUC 0.79, 95% CI = 0.76 to 0.82). The reference model showed comparable performance with all scoring system-based models regarding discrimination (AUC 0.77, 95% CI = 0.74 to 0.80), precision and calibration. See Figures 1 and 2.

## Conclusion

A simple prognostic model, based only on age and GCS, displayed a fairly good prognostic performance in predicting 6-month mortality of ICU-treated patients with moderate-to-severe TBI. The use of more complex scoring systems added little to the prognostic performance.
